# Rapid identification and characterization of genetic loci for defective kernel in bread wheat

**DOI:** 10.1186/s12870-019-2102-6

**Published:** 2019-11-08

**Authors:** Chao Fu, Jiuyuan Du, Xiuling Tian, Zhonghu He, Luping Fu, Yue Wang, Dengan Xu, Xiaoting Xu, Xianchun Xia, Yan Zhang, Shuanghe Cao

**Affiliations:** 1 Institute of Crop Sciences, National Wheat Improvement Center, Chinese Academy of Agricultural Sciences, Beijing, 100081 China; 2 Wheat Research Institute, Gansu Academy of Agricultural Sciences, Lanzhou, 730070 China; 3 International Maize and Wheat Improvement Center, 12 Zhongguancun South Street, Beijing, 100081 China

**Keywords:** Defective kernel, Grain filling, Rapid QTL mapping, SNP array, *Triticum aestivem* L

## Abstract

**Background:**

Wheat is a momentous crop and feeds billions of people in the world. The improvement of wheat yield is very important to ensure world food security. Normal development of grain is the essential guarantee for wheat yield formation. The genetic study of grain phenotype and identification of key genes for grain filling are of great significance upon dissecting the molecular mechanism of wheat grain morphogenesis and yield potential.

**Results:**

Here we identified a pair of defective kernel (Dek) isogenic lines, BL31 and BL33, with plump and shrunken mature grains, respectively, and constructed a genetic population from the BL31/BL33 cross. Ten chromosomes had higher frequency of polymorphic single nucleotide polymorphism (SNP) markers between BL31 and BL33 using Wheat660K chip. Totally 783 simple sequence repeat (SSR) markers were chosen from the above chromosomes and 15 of these were integrated into two linkage groups using the genetic population. Genetic mapping identified three QTL, *QDek.caas-3BS.1*, *QDek.caas-3BS.2* and *QDek.caas-4AL*, explaining 14.78–18.17%, 16.61–21.83% and 19.08–28.19% of phenotypic variances, respectively. Additionally, five polymorphic SNPs from Wheat660K were successfully converted into cleaved amplified polymorphic sequence (CAPS) markers and enriched the target regions of the above QTL. Biochemical analyses revealed that BL33 has significantly higher grain sucrose contents at filling stages and lower mature grain starch contents than BL31, indicating that the Dek QTL may be involved in carbohydrate metabolism. As such, the candidate genes for each QTL were predicated according to International Wheat Genome Sequence Consortium (IWGSC) RefSeq v1.0.

**Conclusions:**

Three major QTL for Dek were identified and their causal genes were predicted, laying a foundation to conduct fine mapping and dissect the regulatory mechanism underlying Dek trait in wheat.

## Background

Wheat (*Triticum aestivum* L.) supplies staple food for more than 35% of population worldwide (http://www.wheatinitiative.org/sites/default/files/attached_file/wheat_facts.pdf). The increase of population, decline of cultivated land and frequent occurrence of extreme climate posed large challenges to meet the consumption of wheat. Identification and mining of genetic loci for grain yield is important to improve the yield potential in wheat. Yield is determined by multiple components, such as grain weight and size. Grain plumpness is an important parameter affecting grain weight and morphological quality [1]. Many genetic loci controlling grain weight have been identified in previous studies [2], whereas genetic analyses on grain plumpness associated with grain filling is rarely reported in wheat. The defective kernel (Dek) mutants in gramineous crops are directly reflected in grain plumpness and usually characterized by shriveled kernel and low grain weight. Thus, identification of genetic loci for Dek is important to improve yield potential and appearance quality. Quite a few Dek mutants were identified in maize [3]. Of them, the genes of *dek1*, *dek35*, *dek38* and *dek39* have been isolated, and all involved in the synthesis and accumulation of starches and/or proteins in the endosperm [4–7]. Many Dek mutants have been generated in wheat, but their genetic information remains largely unknown. SSR markers are characterized with high polymorphisms, good repeatability, co-dominance, and distribution throughout whole genome, and favored for QTL mapping and molecular breeding in wheat [8–10]. With rapid advancements in sequencing technology, high-throughput sequencing or chip-based SNP genotyping platforms have been used for QTL mapping [11–14]. Additionally, the wheat reference genome was assembled and released recently [15]. The availability of high-quality wheat genome data and high-throughput SNP genotyping systems is very important to improve wheat genetics and breeding. We identified a Dek line in a spontaneous variation population and found that its kernels are severely shrunken and sunken. The objective of this study was to rapidly achieve QTL mapping and causal gene prediction for Dek through multiple genotyping systems, wheat reference genome and biochemical analysis.

## Results

### Phenotypic analysis

**Fig. 1:**
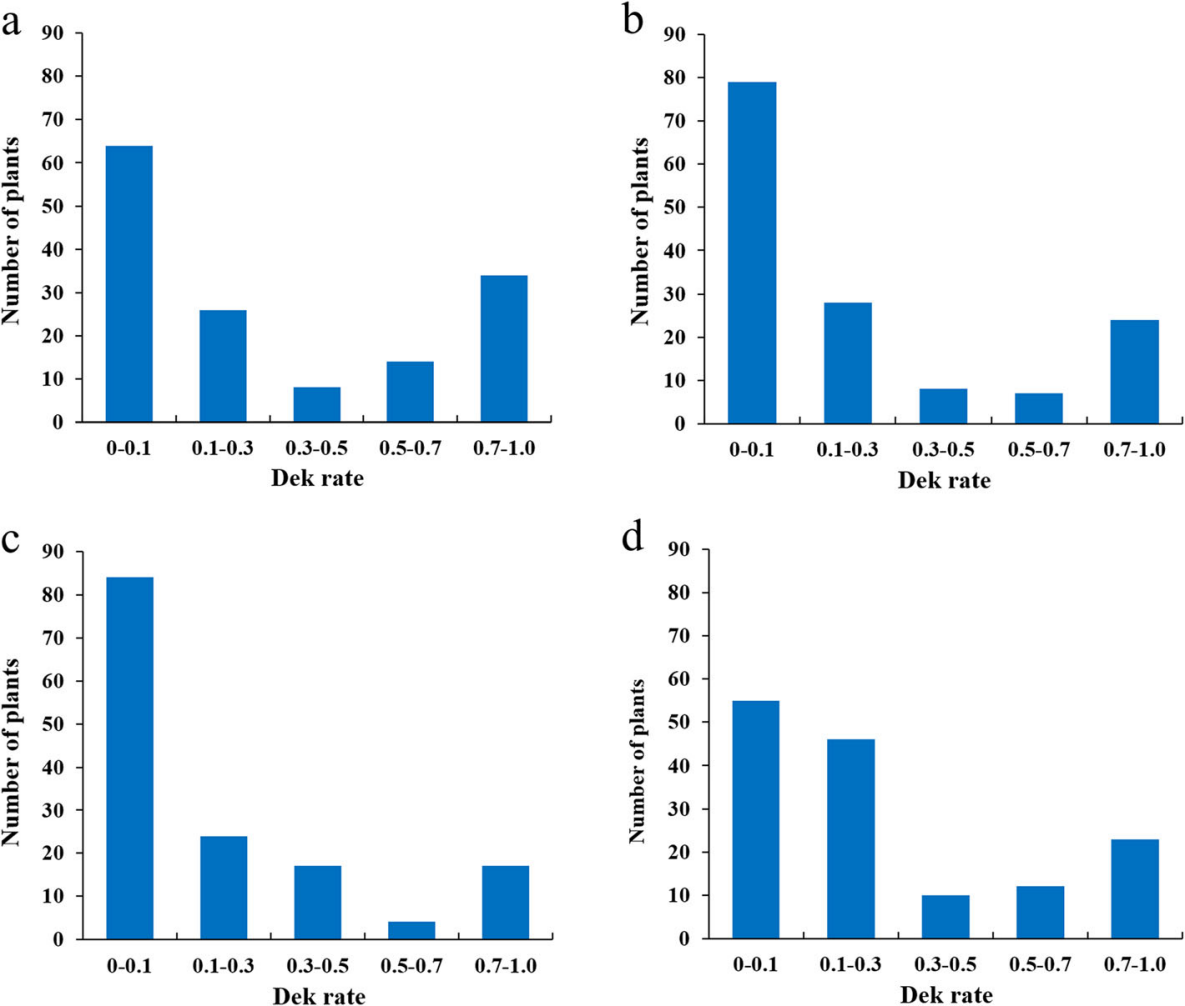
The frequency distributions of Dek rates in Gaoyi 2016–2017 cropping season (**a**), Gaoyi 2017–2018 cropping season (**b**), Xinxiang 2017–2018 cropping season (**c**), and the average data across three environments (**d**)

The kernels of BL31 were normal and plump, whereas the Dek rate of BL33 is 100% in all environments (Additional file 1). The Dek rates in F_2_ and F_2:3_ populations were highly correlated among three environments with correlation coefficients ranging from 0.70 to 0.92 (Table 1). Variance analyses showed that both genotypes (*P* < 0.001) and environments (*P* < 0.05) had significant effects on the target trait (Table 2). The genotype × environment interactions did not reach a statistical significance (*P >* 0.05) (Table 2) and the broad-sense heritability of Dek rate in F_2:3_ across two environments was 0.95, denoting that the Dek rate was mainly determined by genotypes. The Dek rates in the populations showed continuous distribution (Fig. 1, a-d), indicating that Dek is controlled by multiple genetic loci. Additionally, the frequency graph underpinning the phenotypes is abnormal distribution with the “valley - peak” pattern, suggesting the presence of major QTL controlling Dek in the populations [16].

**Table 1:** Statistical analysis of Dek rates for F_2_ and F_2:3_ populations across three environments

Environment	Range	Mean ± SD^a^	Correlation coefficient	
			Gaoyi 2018	Xinxiang 2018
Gaoyi 2017^b^	0~1.00	0.33 ± 0.32	0.70^**^	0.73^**^
Gaoyi 2018^c^	0~1.00	0.24 ± 0.32		0.92^**^
Xinxiang 2018^d^	0~1.00	0.22 ± 0.29		

^a^ SD, standard deviation. ^b^ Gaoyi 2017, Gaoyi, 2016–2017 cropping season. ^c^ Gaoyi 2018, Gaoyi, 2017–2018 cropping season. ^d^ Xinxiang 2018, Xinxiang, 2017–2018 cropping season. ^**^ indicates significance levels at *P <* 0.01

**Table 2:** Analysis of variance of Dek rates for F_2:3_ lines derived from BL33/BL31

Source of variation	Degrees of freedom	Sum of square	Mean square	*F* value
Environment	1	0.097	0.097	5.64^*^
Genotype	145	51.76	0.36	20.68^***^
Genotype × Environment	145	2.21	0.015	0.88
Error	290	5.01	0.017	

^*^ indicates significance levels at *P <* 0.05. ^***^ indicates significance levels at *P <* 0.001

### Chromosomal locations of Dek loci and construction of linkage maps

**Fig. 2:**
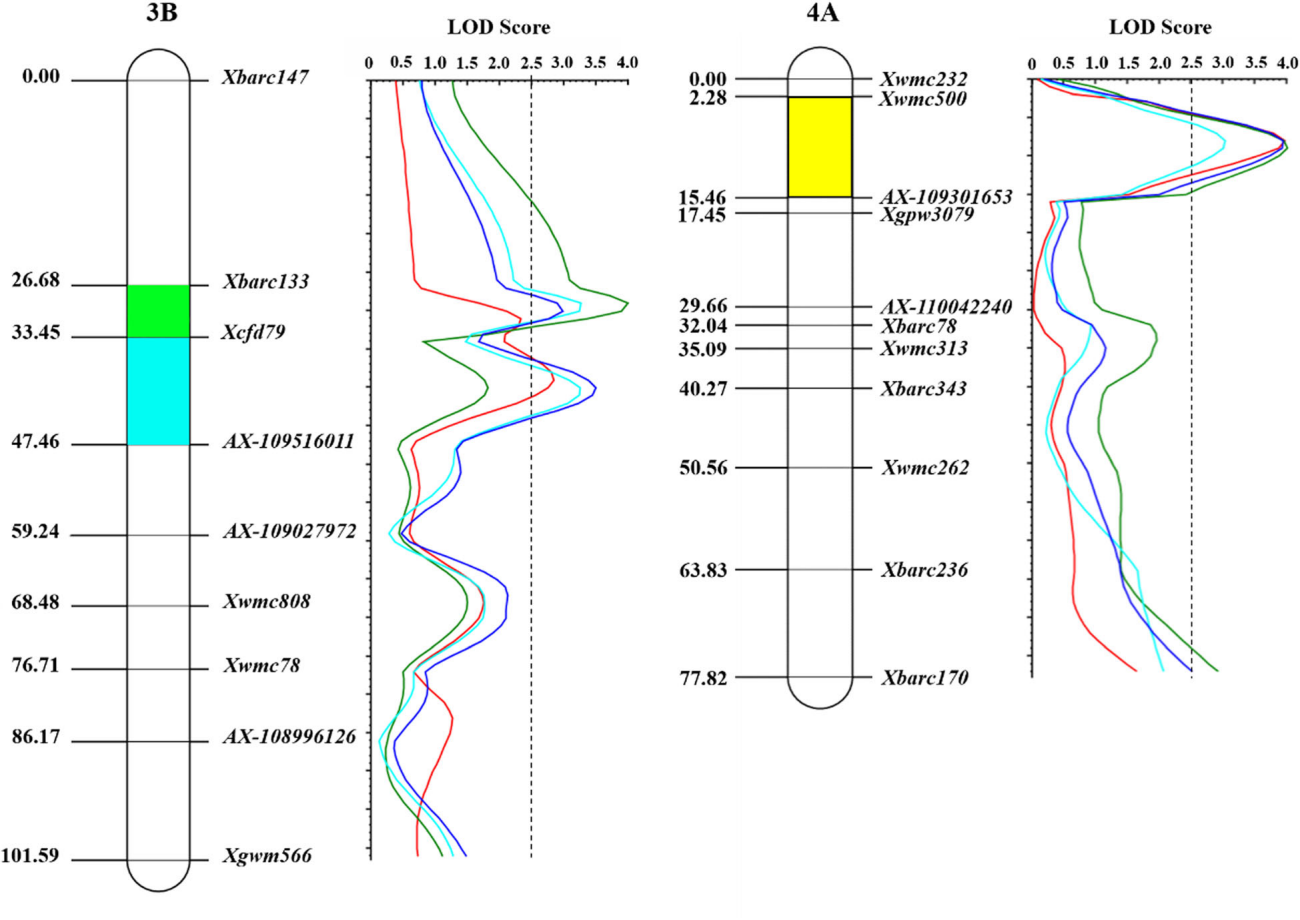
LOD contours for Dek QTL. The genetic regions of *QDek.caas-3BS.1*, *QDek.caas-3BS.2* and *QDek.caas-4AL* are highlighted in *green*, *blue* and *yellow* on chromosomes. LOD contours in Gaoyi 2017, Xinxiang 2018, Gaoyi 2018, and the average are indicated with *red*, *green*, *wathet*, and *deongaree* lines, respectively. LOD threshold of 2.5 is indicated by the dotted vertical line

Theoretically, the more polymorphic markers between isogenic lines one chromosome contains, the higher probability that it harbors QTL for target traits. To rapidly identify the chromosomal locations of Dek QTL, we genotyped the isogenic lines, BL31 and BL33, with contrasting grain phenotypes using the Wheat660K SNP array. Ten chromosomes, 1A, 1B, 2A, 3A, 3B, 4A, 4B, 5A, 6B and 7B, were considered to be potential target chromosomes with Dek QTL based on the frequency distributions of polymorphic SNPs (Additional file 2). To further verify the chromosomal location of Dek QTL, SSR markers from the above target chromosomes were selected to construct genetic map. Totally 783 SSR markers on these chromosomes were selected to construct linkage map for QTL analysis (Additional file 3) [8, 17–23]. Of them, 118 SSR markers displayed polymorphisms between two parents, while only 35 SSRs in chromosomes 3B (13), 4A (19) and 5A (3), showed consistent polymorphisms between two groups of lines with contrasting phenotypes, i.e. 40 defective kernel and 40 normal kernel lines, respectively. Subsequently, the polymorphic SSR markers were used to genotype the entire F_2_ population, and two linkage groups were constructed in chromosomes 3B and 4A, including 6 and 9 SSR markers, respectively (Fig. 2).

### QTL mapping

Based on the resultant linkage groups above and Dek rates in the genetic populations, we performed QTL mapping for Dek traits; three QTL were detected on chromosomes 3B and 4A, designated as *QDek.caas-3BS.1*, *QDek.caas-3BS.2* and *QDek.caas-4AL*, respectively (Table 3; Fig. 2). *QDek.caas-3BS.1* was flanked by *Xbarc133* and *Xcfd79* spanning a genetic interval of 6.77 cM; *QDek.caas-3BS.2* was located between *Xcfd79 and Xwmc808* in an interval of 35.03 cM; *QDek.caas-4AL* was mapped between *Xwmc500* and *Xgpw3079* in a genetic interval of 15.17 cM (Fig. 2). Based on the preliminary mapping above, the physical regions of *QDek.caas-3BS.1*, *QDek.caas-3BS.2* and *QDek.caas-4AL* can be defined by their flanking markers according to IWGSC reference genome RefSeq v1.0 (http://plants.ensembl.org/Triticum_aestivum/Info/Index) [15]. The physical intervals of *QDek.caas-3BS.1*, *QDek.caas-3BS.2* and *QDek.caas-4AL* are 7.60–18.84 Mb, 18.84–32.75 Mb in chromosomes 3BS and 704.34–710.05 Mb in chromosome 4AL. Genetic analyses showed that *QDek.caas-3BS.1*, *QDek.caas-3BS.2* and *QDek.caas-4AL* could explain 14.78 to 18.17%, 16.61 to 21.83% and 19.08 to 28.19% of the phenotypic variances, respectively (Table 3). Each of these QTL determined more than 10% phenotypic variation of Dek in each environment, indicating that they are major QTL, consistent with the “peak - valley” distribution pattern of Dek rate in genetic populations. Additionally, all the alleles governing the formation of Dek came from BL33.

**Table 3:** QTL for Dek trait detected in the F_2_ and F_2:3_ populations across different environments

Environment	QTL	Flanking marker	Physical interval (Mb)^a^	LOD	Add^b^	PVE (%)^c^
Gaoyi 2017^d^	*QDek.caas-3BS.2*	*Xcfd79*-*AX-109516011*	18.84–20.00	2.84	0.13	16.61
	*QDek.caas-4AL*	*Xwmc500*-*AX-109301653*	704.34–705.47	3.98	0.06	28.19
Xinxiang 2018^e^	*QDek.caas-3BS.1*	*Xbarc133*-*Xcfd79*	7.60–18.84	4.00	0.14	18.17
	*QDek.caas-4AL*	*Xwmc500*-*AX-109301653*	704.34–705.47	4.01	0.12	22.60
Gaoyi 2018^f^	*QDek.caas-3BS.1*	*Xbarc133-Xcfd79*	7.60–18.84	3.26	0.14	16.39
	*QDek.caas-3BS.2*	*Xcfd79*-*AX-109516011*	18.84–20.00	3.25	0.17	21.83
	*QDek.caas-4AL*	*Xwmc500*-*AX-109301653*	704.34–705.47	3.03	0.09	19.08
Average	*QDek.caas-3BS.1*	*Xbarc133*-*Xcfd79*	7.60–18.84	2.98	0.11	14.78
	*QDek.caas-3BS.2*	*Xcfd79*-*AX-109516011*	18.84–20.00	3.49	0.13	21.80
	*QDek.caas-4AL*	*Xwmc500*-*AX-109301653*	704.34–705.47	3.95	0.09	23.38

^a^Physical interval defined by physical positions of flanking markers according to IWGSC RefSeq v1.0. ^b^ Add, additive effect; positive values indicate an increasing effect from BL33. ^c^ PVE, phenotypic variance explained. ^d^ Gaoyi 2017, Gaoyi, 2016–2017 cropping season. ^e^ Gaoyi 2018, Gaoyi, 2017–2018 cropping season. ^f^ Xinxiang 2018, Xinxiang, 2017–2018 cropping season

### Marker enrichment in the target regions of QTL

To further narrow down the genetic intervals of target QTL, the polymorphic SNPs identified from the Wheat660K chips were selected according to the above physical regions defined by QTL preliminary mapping. Totally 60 polymorphic SNPs were used to develop site-specific CAPS markers and five of these, *AX-109027972*, *AX-109516011*, *AX-110042240*, *AX-109301653* and *AX-108996126*, were successfully mapped in the linkage groups (Additional file 4; Additional file 5; Additional file 6). Subsequently, *QDek.caas-3BS.2* and *QDek.caas-4AL* were narrowed to the genetic intervals of 14.01 cM and 13.18 cM, respectively (Table 3; Fig. 2). Accordingly, the physical intervals of *QDek.caas-3BS.2* and *QDek.caas-4AL* are reduced to 1.16 Mb and 1.13 Mb, respectively (Table 3). Although the polymorphic loci *AX-108996126* and *AX-110042240* did not further diminish the genetic interval of *QDek.caas-3BS.1* and *QDek.caas-4AL*, they saturated the target regions (Fig. 2). Dek trait displays abnormal development of grain and thus it is usually considered to affect grain weight. To verify this case, we analyzed the effect of the resultant Dek QTL on thousand grain weight (TGW). Compared with BL31, the TGW of BL33 significantly decreased 24.12–28.81% (Additional file 7). QTL analyses based on the linkage maps above showed that only one QTL for TGW, designated as *QTGW.caas-3BS*, was detected between *Xcfd79* and *AX-109027972*, explaining 19.18 to 23.94% of phenotypic variances (Additional file 8). *QTGW.caas-3BS* overlapped *QDek.caas-3BS.2* on the linkage map, suggesting that the Dek QTL *QDek.caas-3BS.2* may affect TGW. No significant effect on TGW was detected in the genetic intervals of the other two QTL, *QDek.caas-3BS.1* and *QDek.caas-4AL*.

### Predication of candidate genes for the Dek QTL

The cloned *dek* genes in maize are involved in the grain growth and development, especially the synthesis and storage of starches and/or proteins in the endosperm [4–7]. Our biochemical analysis also showed that the mature grain starch contents of BL33 were significantly lower than those of BL31 (Additional file 9). In contrast, BL33 had higher sucrose contents and slower sucrose reduction across the filling stages than BL31 (Additional file 10). Accordingly, we assumed that the causal genes for the three QTL are involved in carbohydrate metabolism. In this study, three major QTL was mapped into the genetic intervals of less than 15 cM. The physical region of *QDek.caas-3BS.1* is 11.24 Mb, whereas those of *QDek.caas-3BS.2* and *QDeg.caas-4AL* are only 1.16 and 1.13 Mb, respectively, according to IWGSC RefSeq v1.0 (Table 3). Totally 233 genes were present in the physical intervals of the three QTL (Additional file 11). We found nine genes (Gene ID: *TraesCS3B01G025200, TraesCS3B01G028100, TraesCS3B01G028200, TraesCS3B01G028300, TraesCS3B01G028500, TraesCS3B01G028700, TraesCS3B01G039100, TraesCS3B01G039800*, and *TraesCS4A01G446700*) involving carbohydrate metabolism or grain development in or near the target regions of the Dek QTLs (Additional file 11). To further identify candidate genes for the Dek QTL, the spatio-temporal expression patterns of the nine genes were analyzed using the WheatExp (https://wheat.pw.usda.gov/WheatExp/). Of these, four genes, *TraesCS3B01G025200*, *TraesCS3B01G028700*, *TraesCS3B01G039800*, and *TraesCS4A01G446700*, had an observable expression during grain development (Additional file 12) [24]. Based on functional annotation in IWGSC RefSeq v1.0, the four genes encoded fructose-bisphosphate aldolase (Fba), β-fructofuranosidase, abscisic acid-deficient 4 (ABA4), and sucrose synthase (Sus), respectively, tentatively designated as *TaFba-3B*, *TaBff-3B*, *TaABA4-3B*, and *TaSus-4A*. Their expression patterns were confirmed in the immature grains harvested at three different stages (5, 15, and 25 DAF) using qPCR (Additional file 13). The results showed that all of the above genes expressed in grain and had the lowest expression level at 5 DAF. *TaBff-3B* and *TaSus-4A* was mainly detected at 15 DAF, while *TaFba-3B* and *TaABA4-3B* had the highest expression activity in 25-DAF grains.

## Discussion

### A rapid and cost-effective QTL mapping strategy through multiple genotyping systems in combination with wheat reference genome

**Fig. 3:**
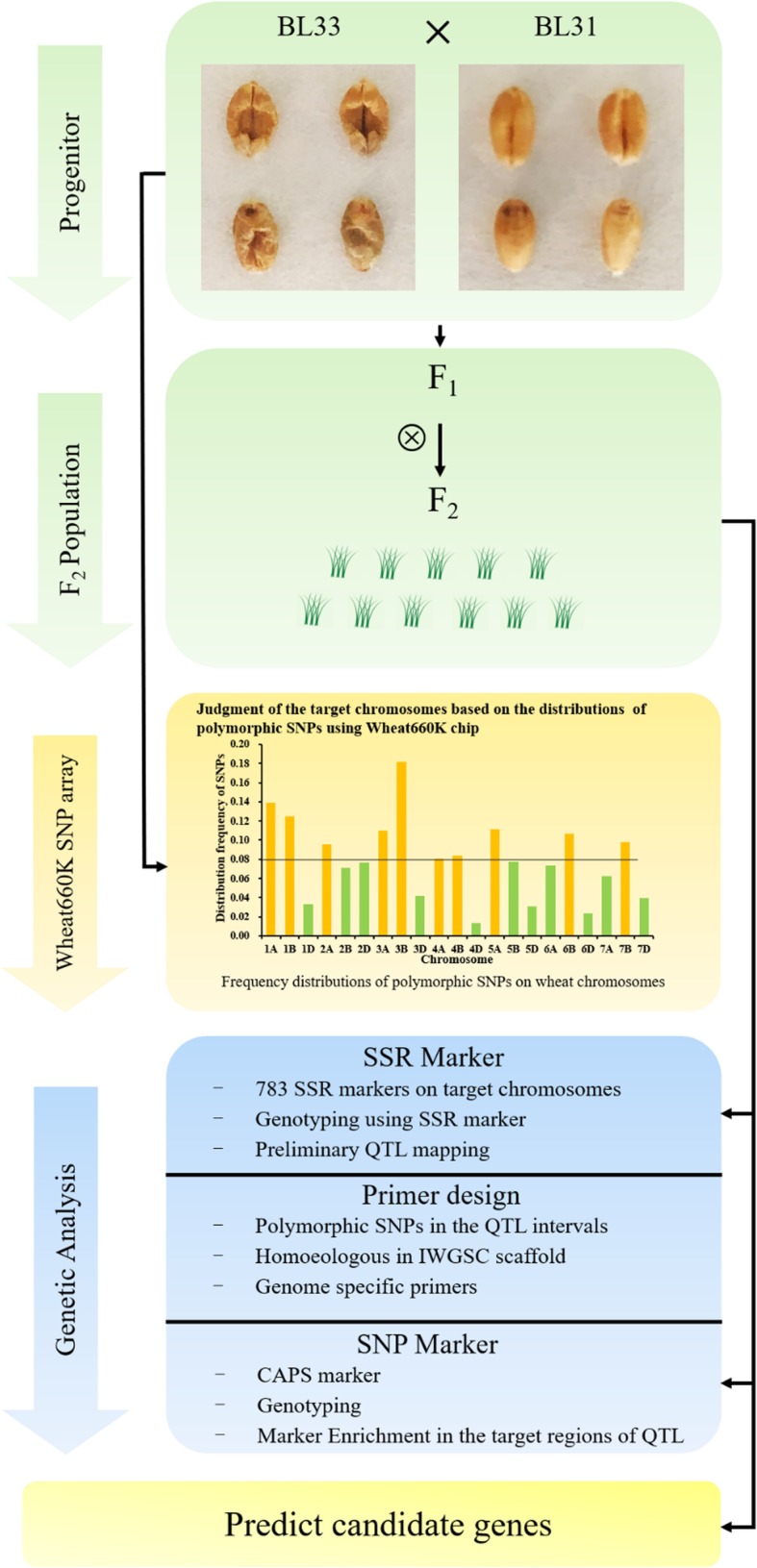
The workflow of rapid and cost-effective QTL mapping for Dek trait. The production of our Fig. 3 referred to the frame of Fig. 1 in Ramirez-Gonzalez et al. [36]

With rapid advances in sequencing technology, more and more wheat varieties were sequenced and a large number of SNPs have been identified [25–27]. Consequently, a few sequencing-based and chip-based SNP genotyping platforms have been developed and used for genetic analyses in wheat [28–30]. Some studies have achieved fine mapping of QTL using SNP chips [31–34]. The chip-based SNP arrays are high-throughput and effective for genetic analyses, but the chips are very expensive. In this study, to rapidly and cost-effectively map QTL for Dek, we only genotyped the isogenic parental lines with contrasting phenotypes using the Wheat660K chip. Firstly, the target chromosomes potentially harboring the QTL for Dek were determined according to the frequency distributions of the polymorphic SNPs. Secondly, it is necessary to construct linkage map for QTL analysis through genotyping genetic populations. However, it is well-known that successful conversion of SNP to the flexible markers, such as KASP and CAPS, is laborious and time-consuming, especially in the hexaploid wheat with three homoeologous subgenomes. Considering high polymorphisms, good repeatability, co-dominance, and distribution throughout whole genome of SSR markers, we harvested the handy SSR markers in the candidate chromosomes above and rapidly constructed linkage maps [8, 35]. Thirdly, we performed genetic mapping for Dek QTL and defined their physical intervals according to IWGSC RefSeq v1.0. Finally, we successfully converted some of the polymorphic SNPs in the physical target regions to CAPS markers and narrowed down the genetic regions of the QTL. In all, we set up a rapid and efficient strategy for QTL mapping through combining high-throughput SNP arrays with cost-effective and flexible SSR markers as well as wheat reference genome (Fig. 3). Of course, a genome-wide linkage map is necessary to find the QTL in all chromosomes. In the future, we intend to construct a recombinant inbred line (RIL) population and achieve genome-wide identification for Dek QTL.

### Comparisons of the Dek QTL with previously reported Dek-related genetic loci

The comparisons of genetic loci for Dek-related traits can not only improve the understanding of genetic effects of the resultant QTL in the present study but also supply with genetic information for QTL fine mapping. *QDek.caas-3BS.1* is linked with *Xbarc133* (Table 3; Fig. 2). Two QTL, *QGfrmax.nfcri-3B* and *QGfd.nfcri-3B*, controlling grain filling rate (GFR) and grain filling duration (GFD), respectively, were linked to *Xgwm533* with a physical distance of 1 Mb from *Xbarc133* [37]. *QTgw.nfcri-3B* for TGW was also linked to *Xgwm533* [37]. *QDek.caas-3BS.1* and *QDek.caas-3BS.2* were identified in adjacent regions and both are linked to *Xcfd79* (Table 3; Fig. 2). A QTL for TGW linked with *Xcfd79* was detected by Groos et al. [38]. Additionally, *QMswscf.acs-3B.1* for water soluble carbohydrates (WSC) of main stem at the early anthesis stage, were linked to *Xcfd79* [39]. *QDek.caas-4AL* is flanked by *Xwmc500* and *AX-109301653* (Table 3). The physical distance between *QDek.caas-4AL* and *Wx-B1* is 16 Mb approximately. So far, many QTL for the contents of starch, protein and amylose and amylopectin, and grain yield have been reported on chromosome 4AL close to *Wx-B1* [40–47]. *QGsc4A*, a QTL for grain starch contents, is closely linked to *Wx-B1* [42]. *Qftsc4A* for total grain starch content and *Qfams4A* for grain amylose content were also linked to *Wx-B1* [46]. To identify the genetic relationship between *QDek.caas-4AL* and *Wx-B1*, the gene-specific functional marker developed by Nakamura et al. [48] was used to genotype the parents, BL31 and BL33. The result showed that no polymorphism was detected between them (Additional file 14), suggesting that *Wx-B1* had little effect on Dek trait.

### Candidate genes analysis

Two candidate genes, *TaFba-3B* and *TaBff-3B*, were detected in *QDek.caas-3BS.1* region, and encoded Fba and β-fructofuranosidases, respectively (Table 4). Fba is one of the nonregulated enzymes comprising the Calvin cycle and controlling carbon fixation and starch synthesis in photosynthetic organs, which can catalyze reversible aldol condensation reaction of dihydroxy acetone phosphate and 3-phosphate glyceraldehyde yielding fructose-1,6-diphosphate [49, 50]. β-fructofuranosidase is a key enzyme for synthesis, metabolism and transportation of sucrose and starch, and can irreversibly catalyze sucrose hydrolysis to glucose and fructose [51, 52]. *TaFba-3B* and *TaBff-3B* probably regulate carbohydrate metabolism and consequently affect Dek phenotype. *TaABA4-3B* was predicted as a candidate gene for *QDek.caas-3BS.2.* ABA4 functions in neoxanthin synthesis [53]. Neoxanthin is necessary for maintaining the integrity of the light harvesting chlorophyll and photosynthetic function of leaves, involving the metabolism of photosynthetic product [54, 55]. *TaSus-4A* may be the causal gene of *QDek.caas-4AL. Sus* deficiency led to the *shrunken-1* mutations in maize [56]. Association analyses showed that the two sucrose synthase genes, *TaSus1* and *TaSus2* on wheat homoeologous chromosome groups 7 and 2, respectively, had significant effects on TGW [57, 58]. In near future, we will identify polymorphic sites of these candidate genes to conduct fine mapping for the target QTL and further determine the causal genes for Dek through wheat transformation experiments.

**Table 4:** Candidate genes of Dek QTLs

QTL	Temporary name	Gene ID	Candidate gene
*QDek.caas-3BS.1*	*TaFba-3B*	*TraesCS3B01G025200*	Fructose-bisphosphate aldolase
	*TaBff-3B*	*TraesCS3B01G028700*	β-fructofuranosidase
*QDek.caas-3BS.2*	*TaABA4-3B*	*TraesCS3B01G039800*	Abscisic acid-deficient 4
*QDek.caas-4AL*	*TaSus-4A*	*TraesCS4A01G446700*	Sucrose synthase

## Conclusions

In the study, we detected three DEK QTLs, *QDek.caas-3BS.1*, *QDek.caas-3BS.2* and *QDek.caas-4AL*, each of which could explain more than 10% phenotypic variation. Additionally, their candidate genes, *TaFba-3B*, *TaBff-3B*, *TaABA4-3B*, and *TaSus-4A*, were predicated according to biochemical analysis and IWGSC RefSeq v1.0 annotation. These findings provide important information for QTL fine mapping and molecular mechanistic dissection underlying Dek in wheat.

## Methods

### Plant materials

A number of Dek lines were generated in Wheat Research Institute, Gansu Academy of Agricultural Science (GAAS). Through multi-generation self-crossing, we identified a pair of isogenic lines, BL31 and BL33, with plump and shrunken mature grains, respectively. Genetic populations comprising 146 F_2_ plants and their corresponding F_2:3_ lines from the cross between BL31 and BL33 were created for QTL mapping.

### Field trials and phenotype evaluation

The F_2_ population and parents were planted at Gaoyi (37°33′N, 114°26′E) in Hebei province during the 2016–2017 cropping season. The F_2:3_ lines were sown at Xinxiang (34°53′N, 113°23′E) in Henan province and Gaoyi during the 2017–2018 cropping season. Field trials were arranged in randomized complete blocks with two replicates at each location. Each plot comprised a single 2 m row with 20 plants and spaced 30 cm apart. Grains were harvested in individual plants or lines. Grain phenotype of F_2_ plants was scored using the percentages of defective kernels (defined as Dek rate) in single plants. For F_2:3_ lines, ten representative plants in each line were selected from the middle part of each plot to figure out Dek rate, then the average Dek rate of each plot was used for subsequent statistical and genetic analysis.

### Preliminary mapping of QTL on target chromosomes using SNP chip

High-quality genomic DNA was extracted from fresh leaves following Guillemaut and Laurence [59]. The Wheat660K SNP array containing 630,517 SNPs, a high-throughput genotyping tool developed by Institute of Crop Sciences, Chinese Academy of Agricultural Sciences (https://wheat.pw.usda.gov/ggpages/topics/Wheat660_SNP_array_developed_by_CAAS.pdf), was used to test two isogenic lines BL31 and BL33 at CapitalBio Corporation (Beijing, China; http://www.capitalbio.com/). The frequency distribution of polymorphic SNPs in each chromosome was analyzed using Microsoft Excel 2016 (https://www.microsoft.com/zh-cn/). The target chromosomes with potential QTL were determined based on the frequency distribution of polymorphic SNPs in each chromosome.

### Genotyping by SSR markers

Polymerase chain reaction (PCR) for SSR markers was performed in a reaction mixture of 15 μl containing 7.5 μl of 2 × *Taq* PCR Mix (Beijing HT-biotech Co., Ltd., Beijing; http://www.ht-biotech.net/), 5 pmol of each primer and 50–100 ng of genomic DNA using the ABI Applied Biosystems Veriti 96 Well Thermal Cycler (Gene Co., Ltd., Shanghai; http://www.genecompany.com/). PCR amplification was performed at 95 °C for 5 min, followed by 35 cycles (94 °C for 30s, 55–60 °C for 30s and 72 °C for 30s) and a final extension at 72 °C for 7 min. The PCR products were separated in 8% polyacrylamide gels at 250 V using the JY 600HE Electrophoretic Apparatus (Beijing Junyi Electrophoresis Co., Ltd., Beijing; http://www.bjjunyi.com/) or 2% agarose gels at 220 V using the DYY-7C Electrophoresis Apparatus (Beijing Liuyi Biotechnology Co., Ltd.; http://www.ly.com.cn/).

### Converting chip-based polymorphic SNPs into CAPS markers

To enrich the target genetic region, polymorphic SNPs between BL31 and BL33, are needed to convert into flexible markers, such as kompetitive allele specific PCR (KASP) and CAPS markers. CAPS technology is an efficient tool to genotype SNPs [60], combining PCR with restriction enzymes digestion to differentiate the target alleles with polymorphic SNPs. The polymorphic SNPs between parents in the target region were identified by alignments with DNAMAN software (https://www.lynnon.com/index.html) for designing chromosome-specific CAPS markers [61]. The primers and restriction enzymes used for CAPS markers were listed in Additional file 4.

### Statistical analysis

The analysis of variance (ANOVA) was conducted using the SAS 9.2 software (http://www.sas.com) with PROC MIXED procedure. Broad-sense heritability (*h*_B_^*2*^) was calculated using the following formula [62]: *h*_B_^*2*^ *= σ*^*2*^_g_ */ (σ*^*2*^_g_ *+ σ*^*2*^_ge_ */ r + σ*^*2*^_ε_ */ re).*

in which, *σ*^*2*^_g_, *σ*^*2*^_ge_ and *σ*^*2*^_ε_ were estimates of genotypes, genotype × environment interaction and residual error variances, respectively; *r* and *e* were replicates per environment and the number of environments, respectively. The correlation coefficients of traits among three different environments were computed using the PROC CORR procedure with the SAS 9.2 software.

### Linkage map construction and QTL analysis

Joinmap 4.0 software (http://www.kyazma.nl) was used for linkage map construction [63] and genetic distances between markers were estimated based on the Kosambi mapping function [64]. QTL analysis was conducted by inclusive composite interval mapping (ICIM) based on the Dek rate using IciMapping 4.0 software (http://www.isbreeding.net) [65, 66]. The logarithm of odds (LOD) threshold score of 2.5 was determined for declaring significant QTL based on 1000 permutations with a type I error of *P* < 0.05. The phenotypic variance explained (PVE) were calculated through stepwise regression to estimate individual QTL effects.

### Assays for grain starch and sucrose contents

Six mature grains were grinded to fine powder in a mortar. The resultant powder (0.1 g) was used to measure starch contents with Starch Assay Kit (Beijing Solarbio Science & Technology Co., Ltd., Beijing, http://www.solarbio.net/). Then, the following formula was used to convert mg/g to mg/grain for scoring the grain starch content: Grain starch content (mg/grain) = starch contents (mg/g) * TGW / 1000.

TGW data was shown in Additional file 7. Six grains of two parents were collected at 5, 10, 15, 20, 25, and 30 days after flowering (DAF) to validate the dynamic trend of grain sucrose contents during the filling stage. The dried grain were grinded to fine powder in mortar, and 0.1 g powder was used to measure grain sucrose contents using Plant Sucrose Assay kit (Beijing Solarbio Science & Technology Co., Ltd., Beijing, http://www.solarbio.net/).

### Quantitative real-time PCR

Total RNA was extracted from fresh grain 5, 15, and 25 days after flowering (DAF) using RNAprep pure Plant Kit (Tiangen Biotech Co., Ltd., http://www.tiangen.com/). Reverse transcription was performed using the reverse transcriptase M-MLV (RNase H^−^) (Takara Biomedical Technology Co., Ltd., http://www.takara.com). The iTaq™ Universal SYBR Green Supermix on CFX Real-Time System (Bio-Rad) was used to perform quantitative real-time PCR (qPCR). The profile of qPCR was as follows: 95 °C for 2 min, followed by 40 cycles of 95 °C for 5 s and 58 °C for 20 s. A final dissociation stage was run to generate a melting curve for judgement of amplification specificity. Quantification of qPCR was determined using the 2^-ΔΔCt^ method [67]. *Elongation factor 1 α* (*EF1α*) gene was used as the internal control to calibrate the expression level of genes of interest. An additional file shows the primers used in qPCR (see Additional file 15).

## Supplementary information

**Additional file 1: Figure S1.** The phenotypes of BL31 (a) and BL33 (b) grains.

**Additional file 2: Figure S2.** The frequency distributions of polymorphic SNPs on 21 wheat chromosomes. The chromosomes with potential Dek QTL are highlighted in *orange*. The frequency threshold of 0.08 is indicated by a horizontal line. Distribution frequency is the percentage of polymorphic SNPs in the total ones on each chromosome.

**Additional file 3: Table S1.** SSR markers used in this study.

**Additional file 4: Table S2.** SNPs used to develop CAPS markers.

**Additional file 5: Figure S3.** Design of CAPS marker for *AX-109027972* (a), *AX-108996126* (b), *AX-109301653* (c), *AX-109516011* (d) and *AX-110042240* (e). The arrows and boxes show the target sites and the positions of restriction sites for the CAPS markers, respectively**.** The underlined forward and reverse primers for CASP markers are labeled at specific target sites.

**Additional file 6: Figure S4.** PCR profile of molecular markers to enrich the target regions of QTL. (a) *AX-109027972*. (b) *AX-108996126*. (c) *AX-109301653*. (d) *AX-109516011*. (e) *AX-110042240*. M, Marker (DL2000DNA ladder in a, b, c and e; 20 bp DNA ladder in d; Takara Bio Company). Target fragments are shown with red arrows*.* Lanes 1 and 2 indicate BL33 and BL31, respectively; lane 3 shows F_1_ plants; lanes 4–10 are F_2_ individual plants.

**Additional file 7: Table S3.** Comparison of TGW between BL31 and BL33**.**

**Additional file 8: Figure S5.** LOD contours obtained by inclusive composite interval mapping (ICIM) analysis for TGW QTL on chromosomes 3B in F_2_ and F_2:3_ populations derived from BL33/BL31. The genetic region of *QTGW.caas-3BS* is highlighted in *red*. LOD contour in Gaoyi 2016–2017 cropping season and Gaoyi 2017–2018 cropping season indicated with *red* and *green* lines, respectively. LOD threshold of 2.5 is indicated by the dotted vertical line.

**Additional file 9: Figure S6.** The comparison of grain starch contents in mature grains between BL31 and BL33.

**Additional file 10: Figure S7.** The change of grain sucrose contents after flowering.

**Additional file 11: Table S4.** Annotated genes in the physical intervals of QTL.

**Additional file 12: Table S5.** Transcriptional patterns of candidate genes in the developing grains.

**Additional file 13: Figure S8.** Transcriptional patterns of four candidate genes in the developing grains.

**Additional file 14: Figure S9.** PCR profile of the *Wx-B1* specific marker. M, Marker (DL2000DNA ladder; Takara Bio Company). Target fragments are shown with red arrows*.* Lane 1 indicates Chinese Spring; lane 2 indicates Guandong107; lane 3 and 4 show BL33 and BL31, respectively.

**Additional file 15: Table S6.** Primers used in quantitative real-time PCR.

## Data Availability

The data sets supporting the results of this article are included within the article and its additional files.

## Abbreviations

AGPP: adenosine diphosphate glucose pyrophosphorylase
ANOVA: analysis of variance
CAPS: cleaved amplified polymorphic sequence
CWI: cell wall invertase
DAF: days after flowering
Dek: defective kernel
Fba: fructose-bisphosphate aldolase
GFD: grain filling duration
GFR: grain filling rate
GWAS: genome-wide association study
ICIM: inclusive composite interval mapping
IWGSC: International Wheat Genome Sequence Consortium
KASP: kompetitive allele specific polymerase chain reaction
LOD: logarithm of odds
PVE: phenotype variation explained
qPCR: quantitative real-time PCR
SNP: single nucleotide polymorphism
SSR: simple sequence repeat
Sus: sucrose synthase
TGW: thousand grain weight
WSC: water soluble carbohydrates
