# Supplementary figures and images for: Rapid identification and characterization of genetic loci for defective kernel in bread wheat
Source: BMC Plant Biol. 2019 Nov 8;19:483. doi: 10.1186/s12870-019-2102-6 (PMC6842267; doi:10.1186/s12870-019-2102-6)

**Additional file 1:** The phenotypes of BL31 (a) and BL33 (b) grains.

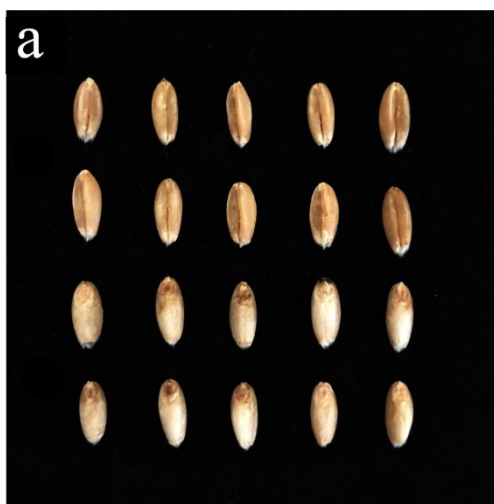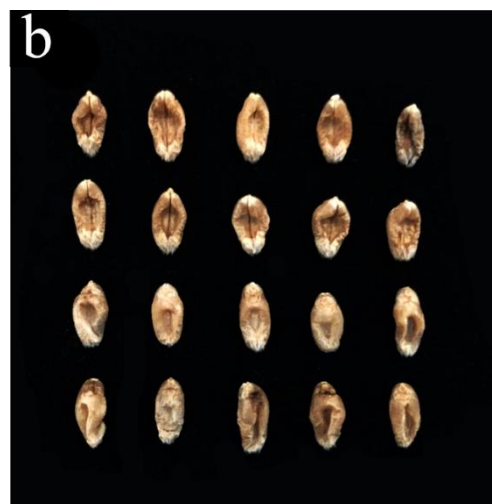

Supplement: Supplementary file 1 — Additional file 1: Figure S1. The phenotypes of BL31 (a) and BL33 (b) grains. [file 12870_2019_2102_MOESM1_ESM.pdf]

**Additional file 9:** The comparison of grain starch contents in mature grains between BL31 and BL33.

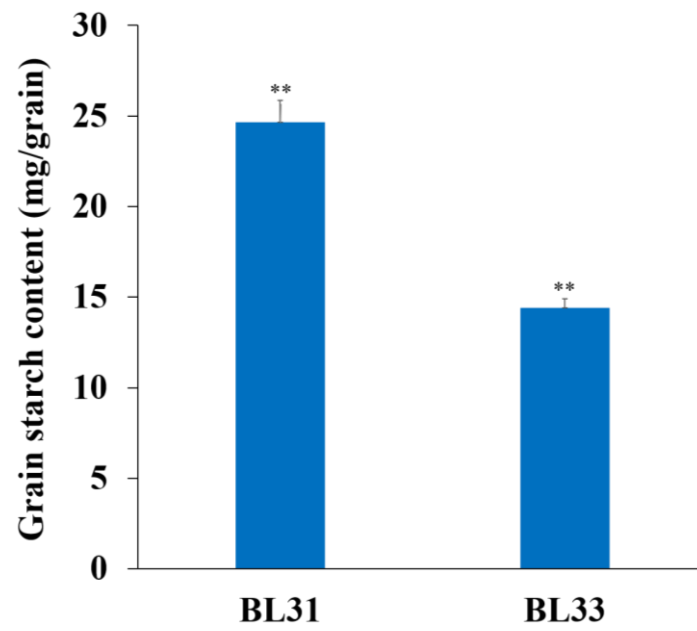

Supplement: Supplementary file 9 — Additional file 9: Figure S6. The comparison of grain starch contents in mature grains between BL31 and BL33. [file 12870_2019_2102_MOESM9_ESM.pdf]

**Additional file 10:** The change of grain sucrose contents after flowering.

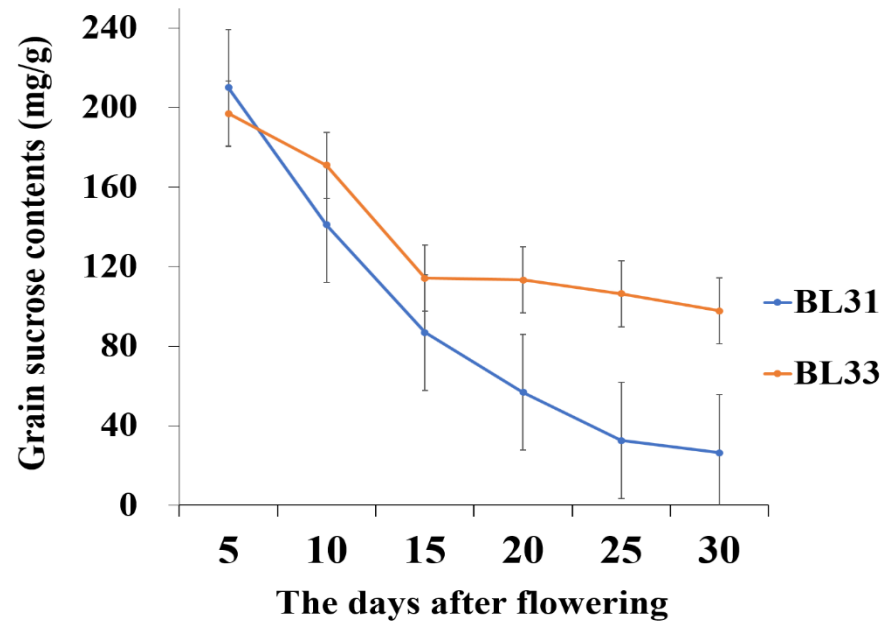

Supplement: Supplementary file 10 — Additional file 10: Figure S7. The change of grain sucrose contents after flowering. [file 12870_2019_2102_MOESM10_ESM.pdf]

**Additional file 13:** Transcriptional patterns of four candidate genes in the developing grains.

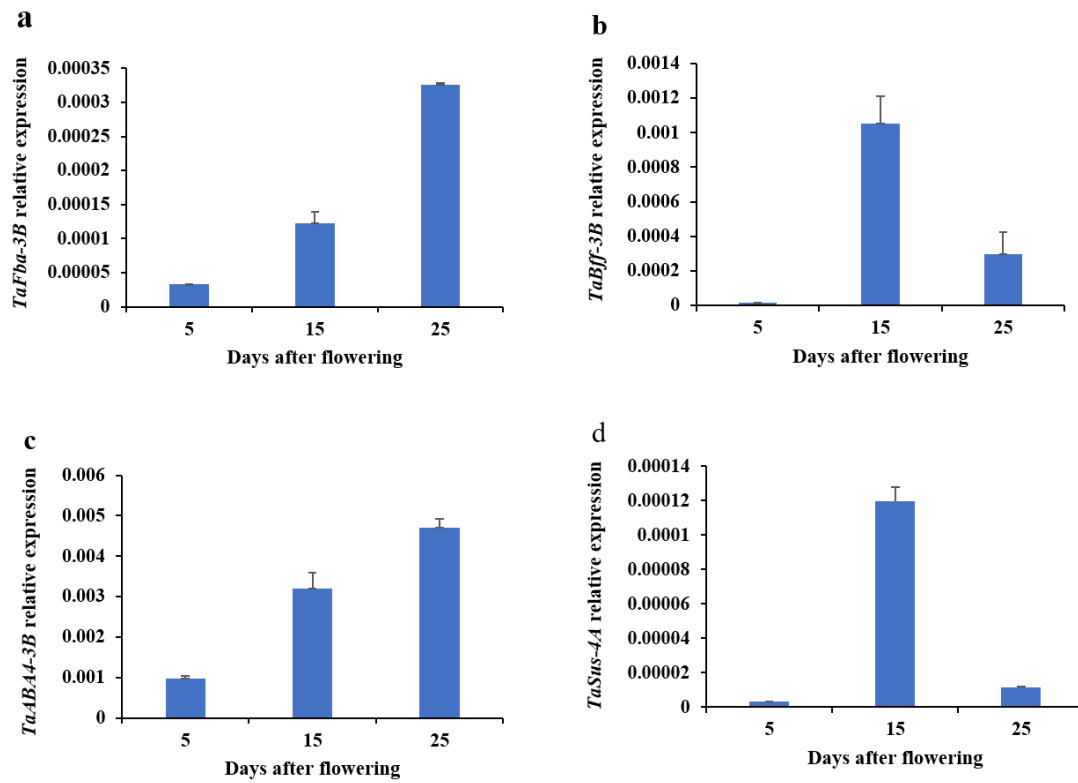

Supplement: Supplementary file 13 — Additional file 13: Figure S8. Transcriptional patterns of four candidate genes in the developing grains. [file 12870_2019_2102_MOESM13_ESM.pdf]
