# Supplementary material for: Rapid identification and characterization of genetic loci for defective kernel in bread wheat
Source: BMC Plant Biol. 2019 Nov 8;19:483. doi: 10.1186/s12870-019-2102-6 (PMC6842267; doi:10.1186/s12870-019-2102-6)

Additional file 2: The frequency distributions of polymorphic SNPs on 21 wheat chromosomes. The chromosomes with potential Dek QTL are highlighted in *orange*. The frequency threshold of 0.08 is indicated by a horizontal line. Distribution frequency is the percentage of polymorphic SNPs in the total ones on each chromosome.

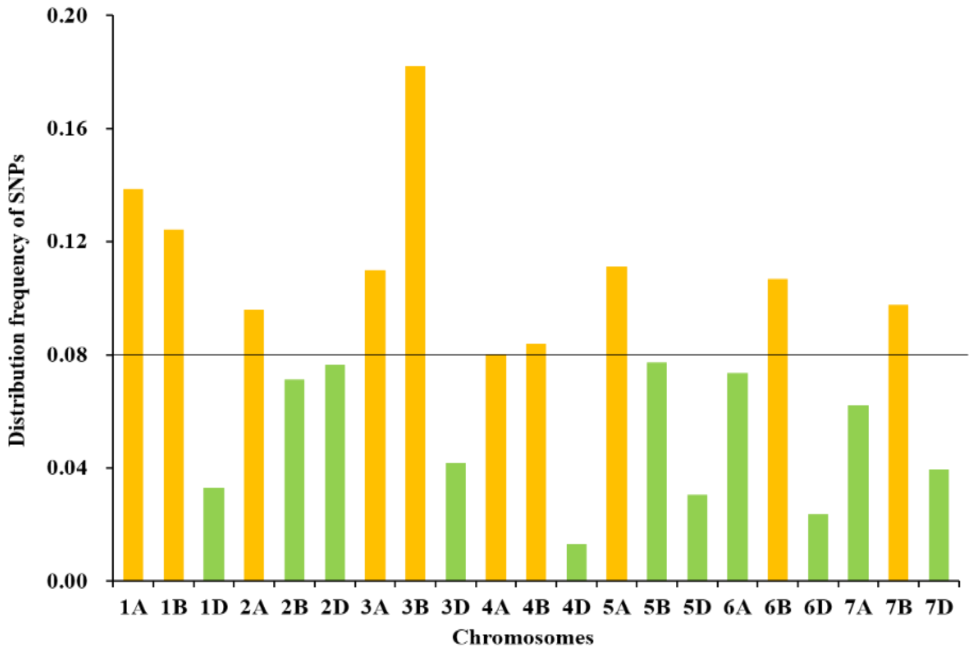

Supplement: Supplementary file 2 — Additional file 2: Figure S2. The frequency distributions of polymorphic SNPs on 21 wheat chromosomes. The chromosomes with potential Dek QTL are highlighted in orange. The frequency threshold of 0.08 is indicated by a horizontal line. Distribution frequency is the percentage of polymorphic SNPs in the total ones on each chromosome. [file 12870_2019_2102_MOESM2_ESM.pdf]
