# Supplementary material for: Rapid identification and characterization of genetic loci for defective kernel in bread wheat
Source: BMC Plant Biol. 2019 Nov 8;19:483. doi: 10.1186/s12870-019-2102-6 (PMC6842267; doi:10.1186/s12870-019-2102-6)
Supplement: Supplementary file 5 — Additional file 5: Figure S3. Design of CAPS marker for AX-109027972 (a), AX-108996126 (b), AX-109301653 (c), AX-109516011 (d) and AX-110042240 (e). The arrows and boxes show the target sites and the positions of restriction sites for the CAPS markers, respectively. The underlined forward and reverse primers for CASP markers are labeled at specific target sites. [file 12870_2019_2102_MOESM5_ESM.pdf]

Additional file 5: Design of CAPS marker for *AX-109027972* (a), *AX- 108996126* (b), *AX-109301653* (c), *AX-109516011* (d) and *AX-110042240* (e). The arrows and boxes show the target sites and the positions of restriction sites for the CAPS markers, respectively**.** The underlined forward and reverse primers for CASP markers are labeled at specific target sites.

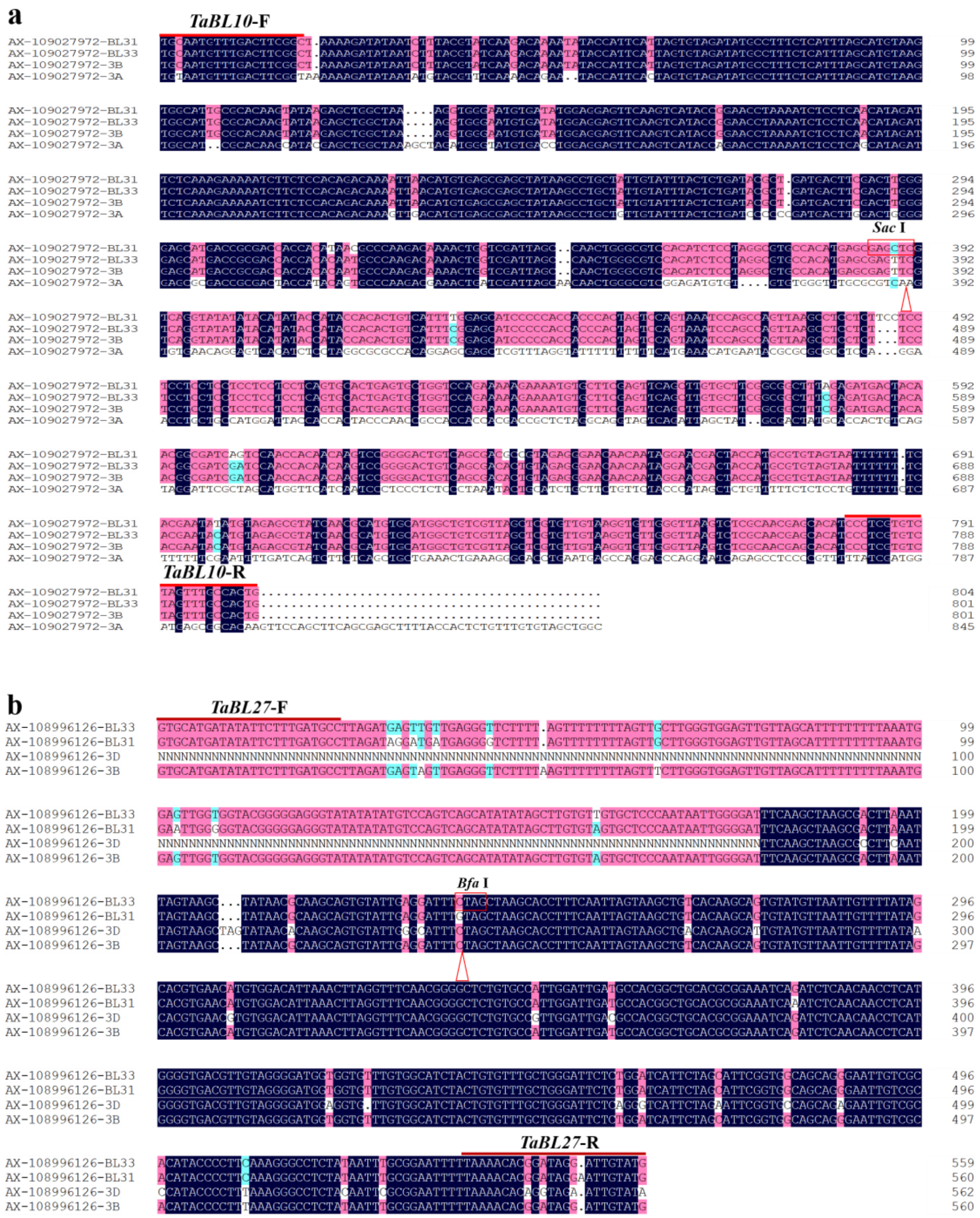

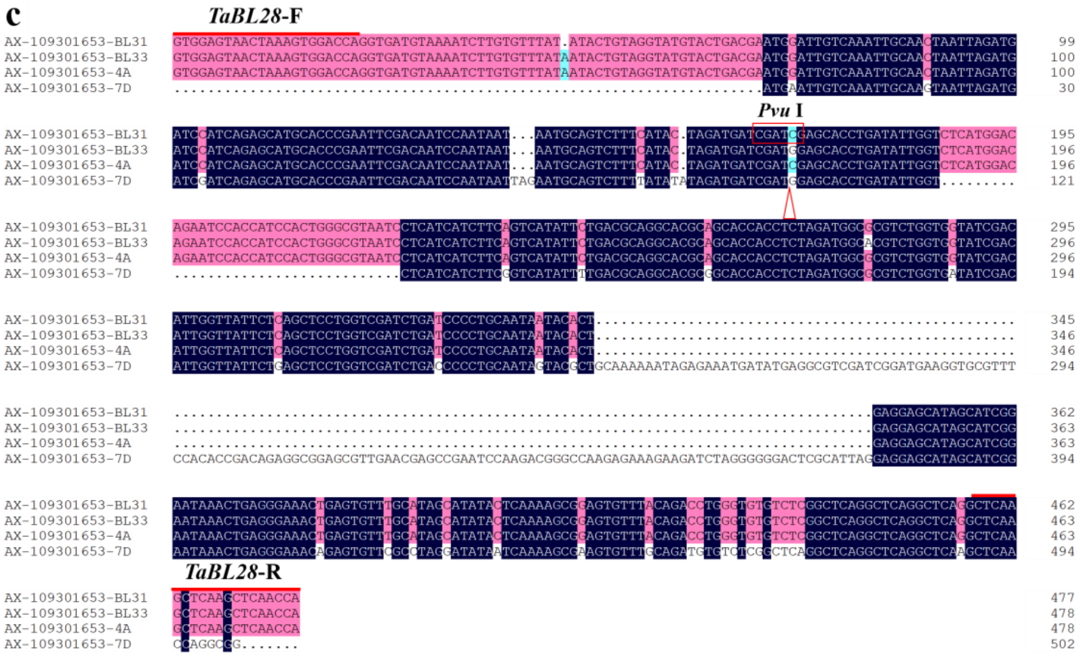
c
TaBL28-F
Pvu I
TaBL28-R
AX-109301653-BL31
AX-109301653-BL33
AX-109301653-4A
AX-109301653-7D
99
100
100
30
195
196
196
121
295
296
296
194
345
346
346
294
362
363
363
394
462
463
463
494
477
478
478
502

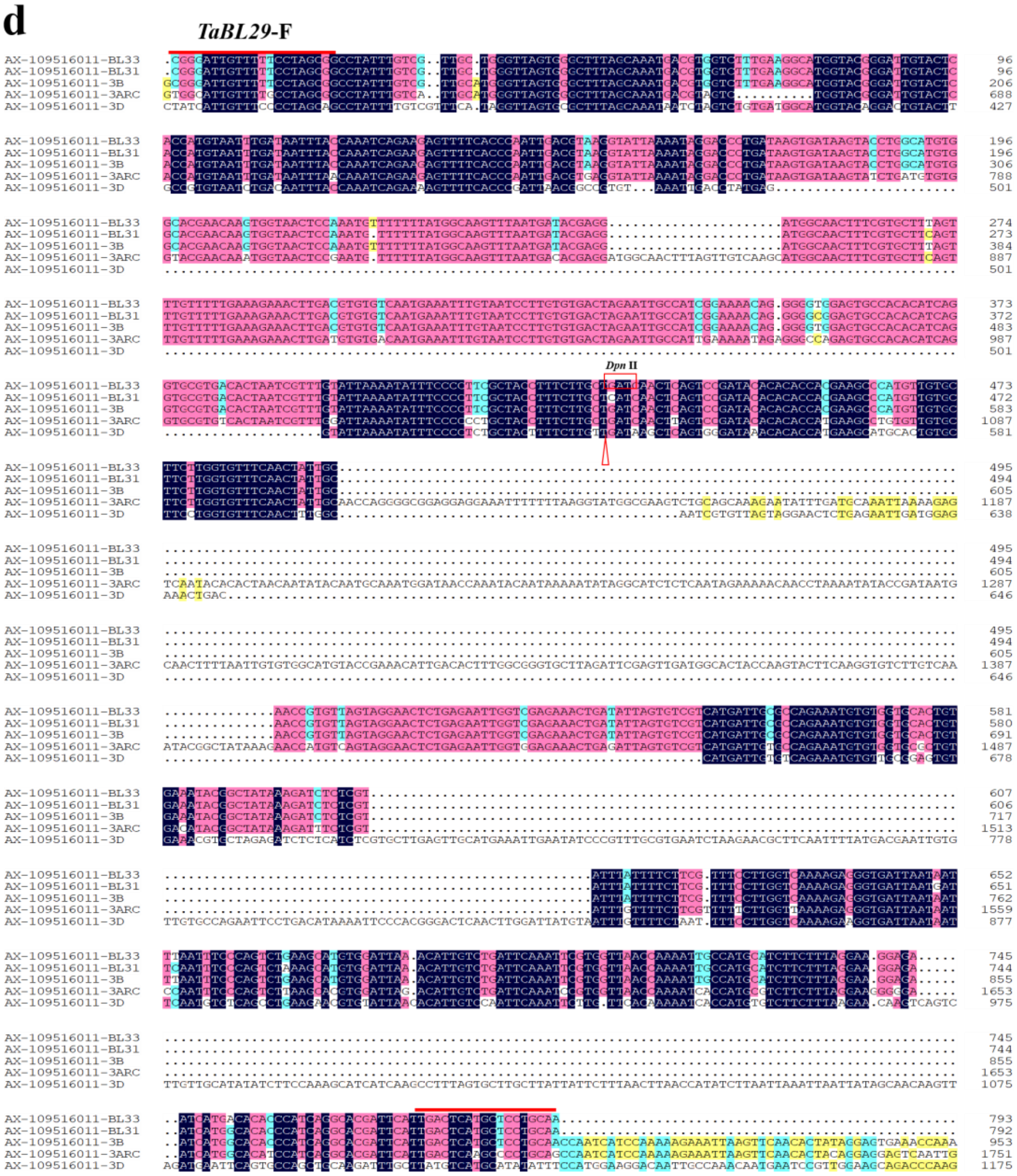
d
TaBL29-F
Dpn II
AX-109516011-BL33
AX-109516011-BL31
AX-109516011-3B
AX-109516011-3ARC
AX-109516011-3D

e

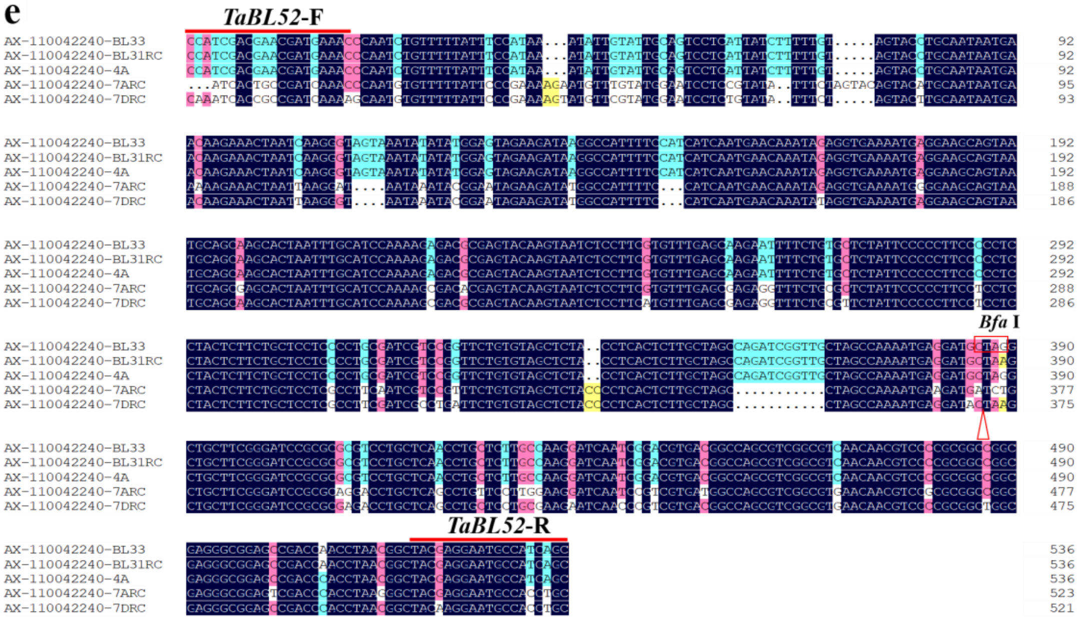

TaBL52-F
AX-110042240-BL33
AX-110042240-BL31RC
AX-110042240-4A
AX-110042240-7ARC
AX-110042240-7DRC
Bfa I
TaBL52-R
