# Supplementary material for: Rapid identification and characterization of genetic loci for defective kernel in bread wheat
Source: BMC Plant Biol. 2019 Nov 8;19:483. doi: 10.1186/s12870-019-2102-6 (PMC6842267; doi:10.1186/s12870-019-2102-6)

Additional file 6: PCR profile of molecular markers to enrich the target regions of QTL. (a) *AX-109027972*. (b) *AX-108996126*. (c) *AX-109301653*. (d) *AX-109516011*. (e) *AX-110042240*. M, Marker (DL2000DNA ladder in a, b, c and e; 20 bp DNA ladder in d; Takara Bio Company). Target fragments are shown with red arrows. Lanes 1 and 2 indicate BL33 and BL31, respectively; lane 3 shows F_1 plants; lanes 4-10 are F_2 individual plants.

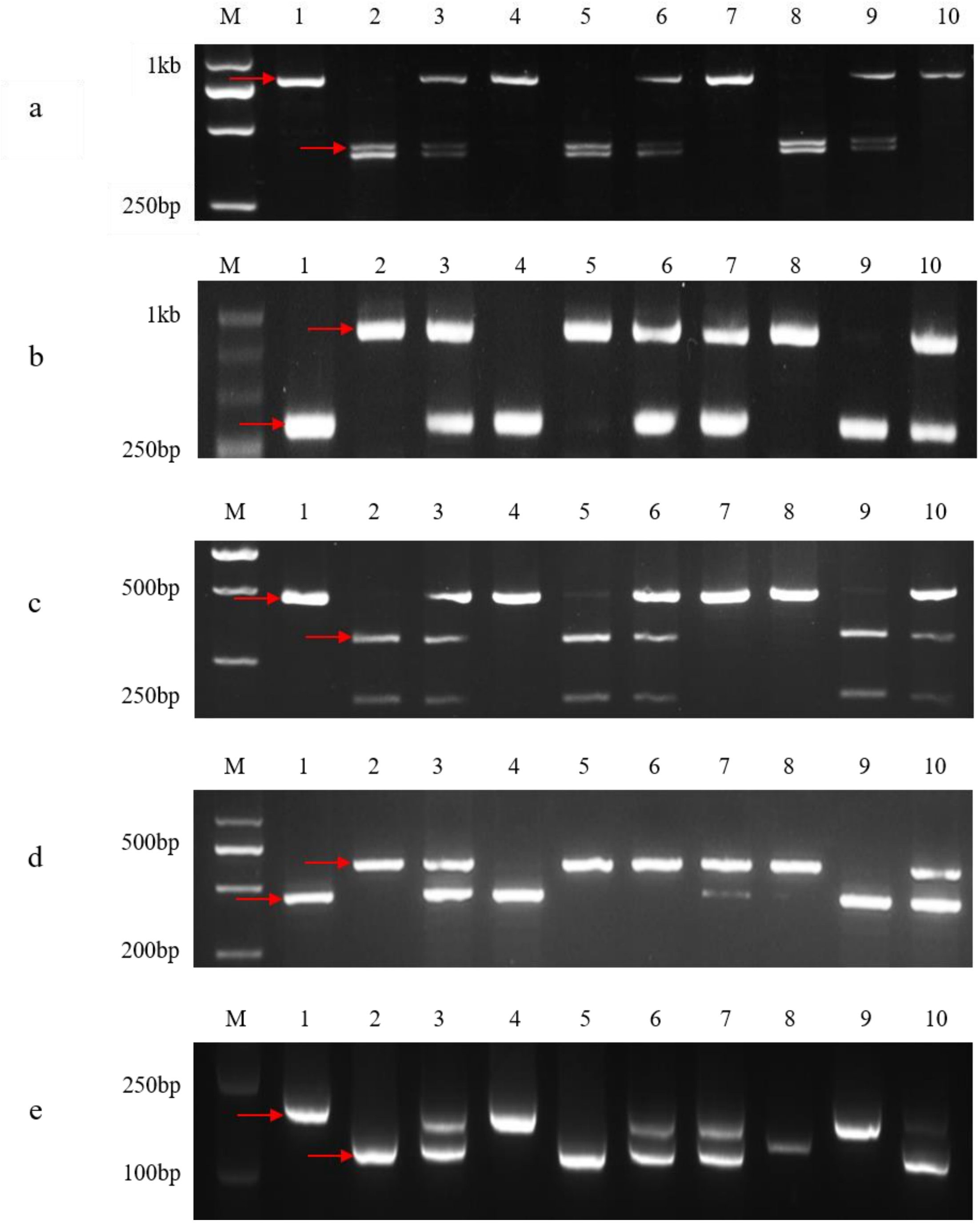

Supplement: Supplementary file 6 — Additional file 6: Figure S4. PCR profile of molecular markers to enrich the target regions of QTL. (a) AX-109027972. (b) AX-108996126. (c) AX-109301653. (d) AX-109516011. (e) AX-110042240. M, Marker (DL2000DNA ladder in a, b, c and e; 20 bp DNA ladder in d; Takara Bio Company). Target fragments are shown with red arrows. Lanes 1 and 2 indicate BL33 and BL31, respectively; lane 3 shows F1 plants; lanes 4–10 are F2 individual plants. [file 12870_2019_2102_MOESM6_ESM.pdf]
