# Supplementary material for: Rapid identification and characterization of genetic loci for defective kernel in bread wheat
Source: BMC Plant Biol. 2019 Nov 8;19:483. doi: 10.1186/s12870-019-2102-6 (PMC6842267; doi:10.1186/s12870-019-2102-6)

Additional file 8: LOD contours obtained by inclusive composite interval mapping (ICIM) analysis for TGW QTL on chromosomes 3B in F_2 and $F_{2:3}$ populations derived from BL33/BL31. The genetic region of *QTGW.caas-3BS* is highlighted in *red*. LOD contour in Gaoyi 2016-2017 cropping season and Gaoyi 2017-2018 cropping season indicated with *red* and *green* lines, respectively. LOD threshold of 2.5 is indicated by the dotted vertical line.

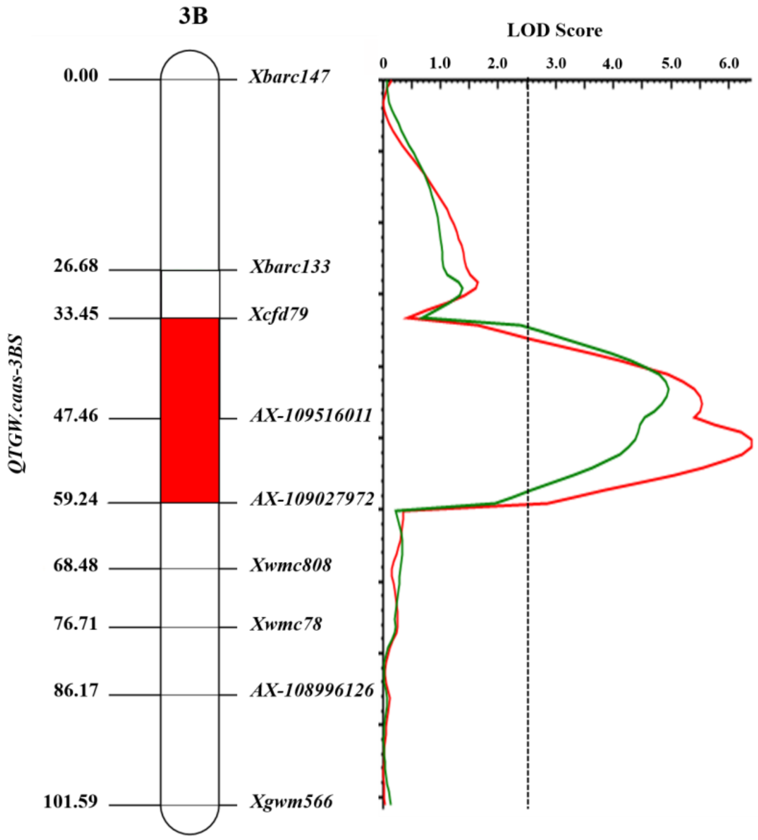

Supplement: Supplementary file 8 — Additional file 8: Figure S5. LOD contours obtained by inclusive composite interval mapping (ICIM) analysis for TGW QTL on chromosomes 3B in F2 and F2:3 populations derived from BL33/BL31. The genetic region of QTGW.caas-3BS is highlighted in red. LOD contour in Gaoyi 2016–2017 cropping season and Gaoyi 2017–2018 cropping season indicated with red and green lines, respectively. LOD threshold of 2.5 is indicated by the dotted vertical line. [file 12870_2019_2102_MOESM8_ESM.pdf]
