# Supplementary material for: Rapid identification and characterization of genetic loci for defective kernel in bread wheat
Source: BMC Plant Biol. 2019 Nov 8;19:483. doi: 10.1186/s12870-019-2102-6 (PMC6842267; doi:10.1186/s12870-019-2102-6)

Additional file 14: PCR profile of the *Wx-B1* specific marker. M, Marker (DL2000DNA ladder; Takara Bio Company). Target fragments are shown with red arrows. Lane 1 indicates Chinese Spring; lane 2 indicates Guandong107; lane 3 and 4 show BL33 and BL31, respectively.

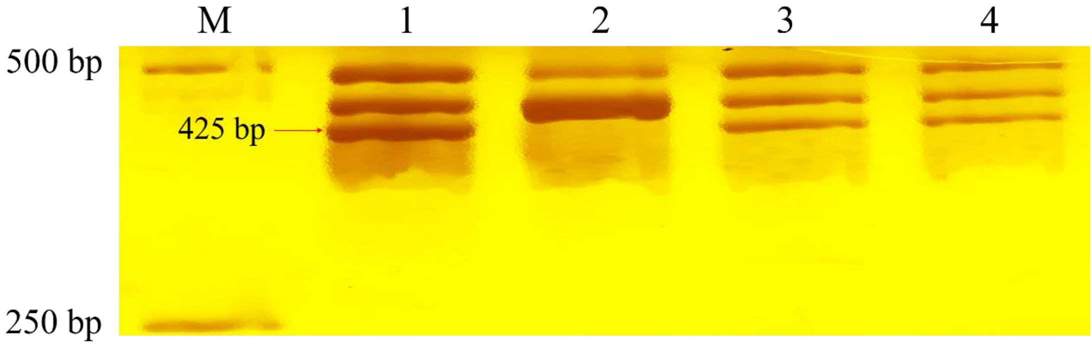

Supplement: Supplementary file 14 — Additional file 14: Figure S9. PCR profile of the Wx-B1 specific marker. M, Marker (DL2000DNA ladder; Takara Bio Company). Target fragments are shown with red arrows. Lane 1 indicates Chinese Spring; lane 2 indicates Guandong107; lane 3 and 4 show BL33 and BL31, respectively. [file 12870_2019_2102_MOESM14_ESM.pdf]
